# Supplementary material for: Stimulation of tumoricidal immunity via bacteriotherapy inhibits glioblastoma relapse
Source: Nat Commun. 2024 May 18;15:4241. doi: 10.1038/s41467-024-48606-5 (PMC11102507; doi:10.1038/s41467-024-48606-5)
Supplement: Supplementary file 1 — Supplementary Information [file 41467_2024_48606_MOESM1_ESM.pdf]

Supplementary Information:

Stimulation of Tumoricidal Immunity via Bacteriotherapy Inhibits Glioblastoma Relapse

Yulin Zhang[1,2,3,10], Kaiyan Xi[1,2,4,10], Zhipeng Fu[3,10], Yuying Zhang[5], Bo Cheng[6], Fan Feng[1,2], Yuanmin Dong[3], Zezheng Fang[1,2], Yi Zhang[1,2], Jianyu Shen[1,2], Mingrui Wang[1,2], Xu Han[1,2], Huimin Geng[1,2], Lei Sun[7], Xingang Li[1,8,9], Chen Chen[3], Xinyi Jiang[3], Shilei Ni[1,2]

[1]Department of Neurosurgery, Qilu Hospital, Cheeloo College of Medicine, Shandong University, 107 Wenhua Xi Road, Jinan, 250012, Shandong, China

[2]Institute of Brain and Brain-Inspired Science, Shandong University, 107 Wenhua Xi Road, Jinan, 250012, Shandong, China

[3]Key Laboratory of Chemical Biology (Ministry of Education), Department of Pharmaceutics, School of Pharmaceutical Sciences, Cheeloo College of Medicine, Shandong University, 44 Wenhua Xi Road, Jinan, 250012, Shandong, China

[4]Department of Pediatrics, Qilu hospital, Cheeloo College of Medicine, Shandong University, 107 Wenhua Xi Road, Jinan, 250012, Shandong, China

[5]Department of Obstetrics, The Second Hospital, Cheeloo College of Medicine, Shandong University, No. 247 Beiyuan Road, Jinan 250033, Shandong, China

[6]Department of Radiation Oncology, Qilu Hospital affiliated to Shandong University, 107 Wenhua Xi Road, Jinan, 250012, Shandong, China

[7]Department of Endocrinology, Qilu Hospital, Cheeloo College of Medicine, Shandong University, 107 Wenhua Xi Road, Jinan, Shandong 250012, China

[8]Jinan Microecological Biomedicine Shandong Laboratory, Jinan 250117, Shandong, China

[9]Shandong Key Laboratory of Brain Function Remodeling, Jinan 250117, Shandong, China

[10]These authors contributed equally: Yulin Zhang, Kaiyan Xi, Zhipeng Fu.

These authors jointly supervised this work: Chen Chen, Xinyi Jiang, Shilei Ni

Shilei Ni: nishilei@sdu.edu.cn

Xinyi Jiang: xinyijiang@sdu.edu.cn

Chen Chen: chenchen27@sdu.edu.cn

Supplementary Methods

Strain construction of VNP20009 Δ*invA:sfgfp*

1 *invA* knockout replacement repair homology arm construction

1.1 Homology arm fragment and *sfgfp* gene amplification

The upstream and downstream homology arm sequences of *invA* were amplified using VNP20009 DNA as template with primers invA-19-F, invA-sfgfp-R, invA-sfgfp-F and invA-19-R. Amplification of *sfgfp* fragments with primers sfgfp-invA-F/R, using the *sfgfp* plasmid as a template (Supplementary Fig. 29).

Amplification system:

2×pfu PCR mix	25μl
Primer P1(10μM)	2μl
Primer P2(10μM)	2μl
Template	2μl
ddH_2O	19μl
Total	50μl

Amplification condition: 94°C 5min 30Cycle (94°C 30sec, 55°C 30sec, 72°C 40sec)

1.2 Seamless cloning

The three amplified fragments were seamlessly cloned according to the following system.

5×infusion cloing mix	2μl
Up-Recycled fragments	2μl
Sfgfp-Recycled fragments	2μl
Down-Recycled fragments	2μl
puc19[*Eco*RI-*Hin*dIII] linear carrier	2μl
Total	10μl

After mixing, leave on ice for 30min, transform *E. coli* DH5α competent cells, spread Amp/IPTG/x-gal plate, and incubate at 37°C to pick the white fluorescent clone for sequencing. The sequenced clone was named invA(up-sfgfp-down)-puc19.

1.3 Repair homology arm amplification

The correctly sequenced invA(up-sfgfp-down)-puc19 plasmid was used as a template with primers invA-up-F/invA-down-R. The primers were used to amplify the repaired homology arm of the *invA* knockout substitution according to the following system.

2×superpfu PCR mix	25μl
invA-up-F(10μM)	2μl
invA-down-R(10μM)	2μl
invA(up-sfgfp-down)-puc19	1μl
ddH_2O	20μl
Total	50μl

Amplification conditions: 94°C 5min 32Cycle (94°C 30sec, 55°C 30sec, 68°C 40sec) 10°C hold on

2 CRISPR/Cas9 *invA* knockout replacement strain screening

2.1 Preparation of VNP20009 competent cells

1) Activated culture of VNP20009 at 37°C;
2) Inoculate the monoclonal to 5ml LB liquid medium;
3) The next day it was transferred to 50 ml LB liquid medium and centrifuged to collect the bacteria at an OD growth rate of about 0.8;
4) The bacteria were washed three times with 10% glycerol and finally resuspended with 2ml 10% glycerol as the prepared competent cells.

2.2 Electrotransformation of plasmids containing pCas9

Add 10μl of plasmid to the prepared competent cells and place them on ice for 5min, electrotransform them at 2500V, add 1ml of LB medium, and incubate them at 37℃ for 1h before spreading the Kan-resistant plate.

2.3 Transformation of sgRNA and repair of homology arms

Recovered *invA-sfgfp* repair homology arm and prepared sgRNA plasmid were electrotransformed into prepared Cas9 VNP20009 competent cells, and Kan/Tc plates were coated to screen for integrating strains.

2.4 Gene identification of VNP20009 Δ*invA:sfgfp*

The primers invA-up-F/invA-down-R and invA-ter-F/R were used to identify wild bacteria and integrating bacteria (Supplementary Fig. 30). After identification, the tool plasmid was eliminated to obtain VNP20009 Δ*invA:sfgfp*. The editing bacteria containing Cas9 plasmid were also saved for use in the next editing step.

Strain construction of VNP20009 Δ*invA:sfgfp glms:FNR-invA*

1 *FNR-invA* insertion repair homology arm construction

1.1 Amplification of insertion site homology arms and insertion gene fragments

The upstream and downstream homology arms of *glms* were amplified with primers glms-19-F/glms-FNR-R and glms down-invA-F/glms-19-R, respectively, using VNP20009 DNA as a template. The *FNR* promoter sequence was amplified using the *FNR* promoter vector, and the *invA* gene fragment for insertion was amplified using invA-FNR-F/invA-his-R (Supplementary Fig. 31).

Amplification system:

2×pfu PCR mix	25μl
Primer P1(10μM)	2μl
Primer P2(10μM)	2μl
Template	2μl
ddH_2O	19μl
Total	50μl

Amplification conditions: 94°C 5min 30Cycle (94°C 30sec, 55°C 30sec, 72°C 40sec) 10°C hold on

1.2 Seamless clonal joining of homologous arm fragments and insertion genes

The amplified fragments were purified from PCR products and then seamlessly clonally ligated according to the following system conditions.

5×infusion cloing mix	2μl
Up-Recycled fragments	2μl
invA-Recycled fragments	2μl
FNR-Recycled fragments	1μl
down-Recycled fragments	2μl
puc19[*Eco*RI-*Hin*dIII] linear carrier	1μl
Total	10μl

After mixing and placing on ice for 30 min, *E. coli* DH5α competent cells were transformed and cultured on Amp/IPTG/x-gal coated plates, followed by picking white single clones for sequencing. The correctly sequenced clone was named glms-Up-FNR-invA-down-pUC19.

1.3 Preparation of repair homology arms for *FNR-invA* insertion

Repair of homology arm fragments by amplification with primers glms-up-F/glms-down-R using the correctly sequenced glms-Up-FNR-invA-down-pUC19 plasmid as a template.

2 CRISPR/Cas9 *invA* insertion strain screening

2.1 Preparation of VNP20009 Δ*invA:sfgfp* pCas9 competent cells as described above.

2.2 Transformation of sgRNA and repair of homology arms

Recovered *glms-FNR-invA* repair homology arm and prepared glms sgRNA plasmid were added to prepared VNP20009 Δ*invA: sfgfp* pCas9 competent cells, placed on ice for 5 min, transformed by 2500V. The integrated strains were screened using coated Kan/Tc plates after adding 1 ml of LB medium and shaking the culture for 1h.

2.3 Gene identification of VNP20009 Δ*invA:sfgfp glms:FNR-invA*

The primers glms-up-F/glms-down-R were used to identify wild bacteria and integrating bacteria (Supplementary Fig. 32). After identification, the tool plasmid was eliminated to obtain VNP20009 Δ*invA:sfgfp glms:FNR-invA*.

Construction of *LysE* gene expression vector and transformation screening

1 Seamless cloning

The *LysE* fragment amplification product and the pBAD plasmid linearisation digest product were seamlessly clonally ligated according to the following system conditions.

5×infusion cloing mix	2μl
LysE Recycled fragments	6μl
pBAD *Eco*RI digested vector fragments	2μl
Total	10μl

After mixing and placing on ice for 30 min, *E. coli* DH5α competent cells were transformed and incubated on Amp plates at 37°C to pick monoclonal identification.

2 PCR identification

Pick the monoclonal to 10 μl of aseptic water and mix well, then take 0.5 μl as a template, according to the following system for identification. Then positive clonal plasmids were picked and sent for sequencing, and the correctly sequenced plasmid was named *pBAD-LysE*.

2×Taq PCR mix	10μl
pBAD-F(10μM)	0.5μl
pBAD-R(10μM)	0.5μl
bacterial solution	0.5μl
ddH$_2$O	8.5μl
Total	20μl

Amplification conditions: 94°C 5min 30Cycle (94°C 30sec, 55°C 30sec, 72°C 1min) 10°C hold on.

3 Electrotransformation of *pBAD-LysE*

Preparation of VNP20009 Δ*invA:sfgfp glms:FNR-invA* competent cells as described above. Take 1μl of plasmid and add it to the prepared competent cells and place it on ice for 5min, electrotransform it at 2500V, add 1ml of LB medium, recover the culture at 37°C for 1h and then spread the Amp resistance plate.

4 Gene identification of VNP20009 Δ*invA:sfgfp glms:FNR-invA pBAD-LysE*

The primers pBAD-F/pBAD-R were used to identify wild bacteria and integrating bacteria. Positive clones were sent for sequencing and the final positive clone identified was VNP20009 Δ*invA:sfgfp glms:FNR-invA pBAD-LysE* (Supplementary Fig. 33).

The primer sequences involved above are shown in Supplementary Table 3.

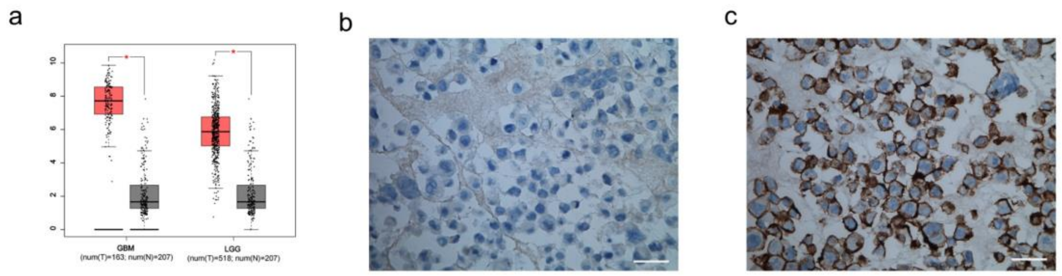

Supplementary Figure 1. (a) mRNA expression levels of CD44 in GBM and low-grade glioma (LGG) analyzed by GEPIA database. The statistical comparisons were performed with the one-way analysis of variance (ANOVA) with Tukey's post hoc test. (b) Immunohistochemical staining showing the expression levels of CD44 in NHA cells. ($n = 3$ independent experiments). (c) Immunohistochemical staining showing the expression levels of CD44 in QL01# GBM cells. ($n = 3$ independent experiments). The scale bar is 10 μm.

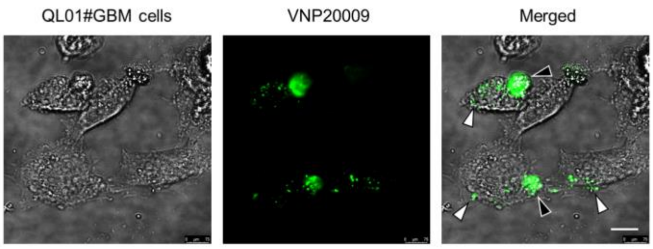

Supplementary Figure 2. Formation of SCVs (black arrows) inside GBM cells after SDV invasion. (n = 3 independent experiments). The scale bar is 10 μm.

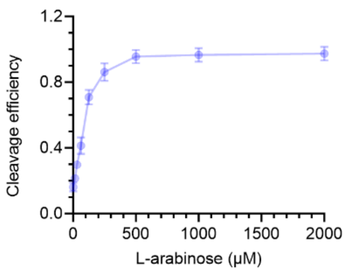

Supplementary Figure 3. Efficiency curves demonstrating SDV cleavage at various L-arabinose concentrations. ($n = 6$ independent experiments). Data are presented as the mean ± S.D. Source data are provided as a Source Data file.

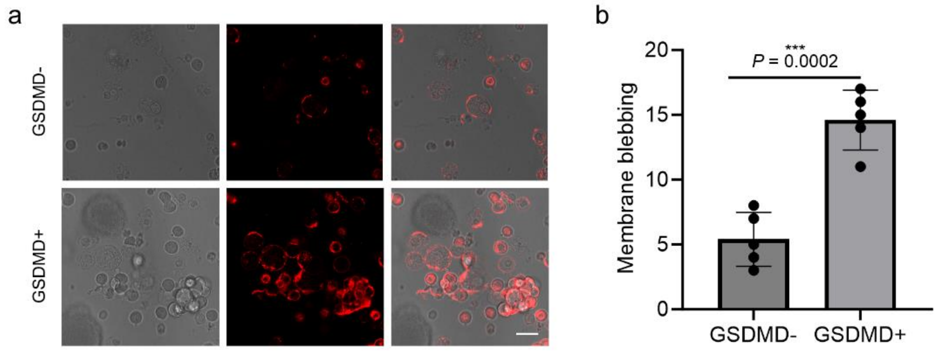

Supplementary Figure 4. Fluorescence staining (a) and statistical analysis (b) of cell membrane vacuole formation in GSDMD high versus low expressing QL01#GBM cells. (n = 5 independent experiments). The scale bar is 10 μm. The statistical comparisons in (b) were performed with two-tailed, unpaired Student's t tests, with asterisks indicating significant differences. Source data are provided as a Source Data file.

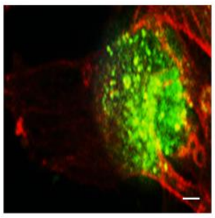

Supplementary Figure 5. Formation of SCVs after IASNDS treatment in QL01#GBM cells. (*n* = 3 independent experiments). The scale bar is 1 μm.

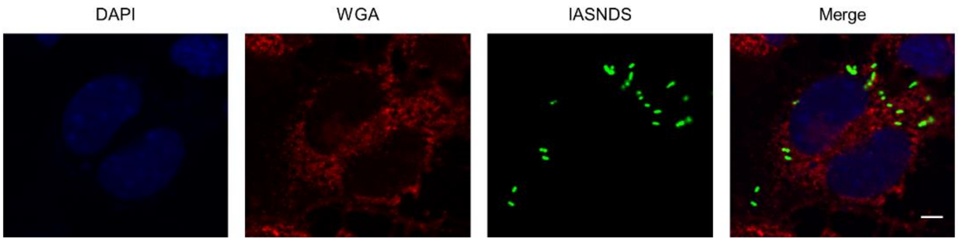

Supplementary Figure 6. Bacterial invasion into QL01#GBM cells after 2 hours of IASNDS treatment (green), with no SCV formation. ($n = 3$ independent experiments). The scale bar is 1 μm.

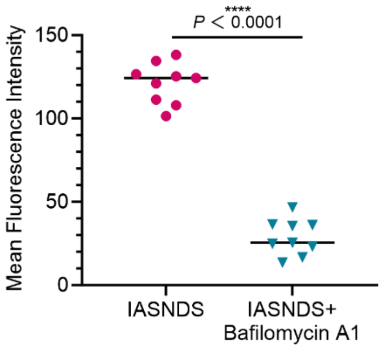

Supplementary Figure 7. Mean fluorescence intensity of QL01#GBM cells after IASNDS-induced autolysis following Bafilomycin A1 inhibition of lysosomal function. ($n = 9$ images from three independent experiments). The statistical comparisons were performed with two-tailed, unpaired Student's *t* tests, with asterisks indicating significant differences. (exact *P* value: $P = 1.0382E\text{-}11$); ****$P < 0.0001$. Source data are provided as a Source Data file.

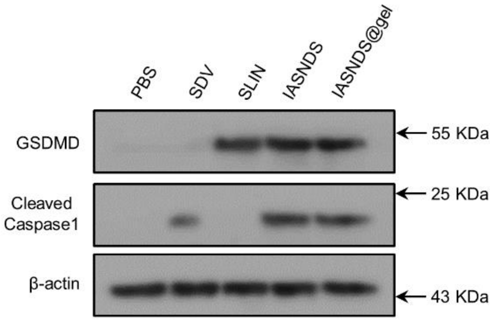

Supplementary Figure 8. Western blot confirming increased cellular GSDMD expression in the SLIN treatment group, along with enhanced cleaved caspase 1 expression due to the SDV component. ($n = 3$ independent experiments).

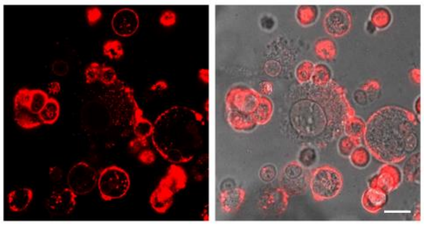

Supplementary Figure 9. Confocal microscopy images showing cellular pyroptosis after 24 hours of IASNDS treatment (Red Membrane) in QL01#GBM cells. ($n = 3$ independent experiments). The scale bar is 5 μm.

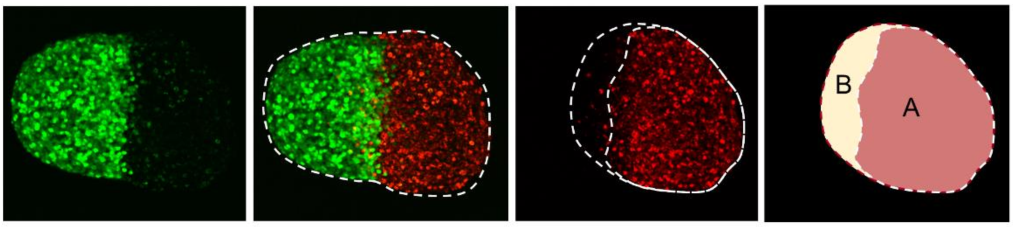

Supplementary Figure 10. Statistical analysis approach for the 3D invasion experiment, with calculation of the invasion rate.

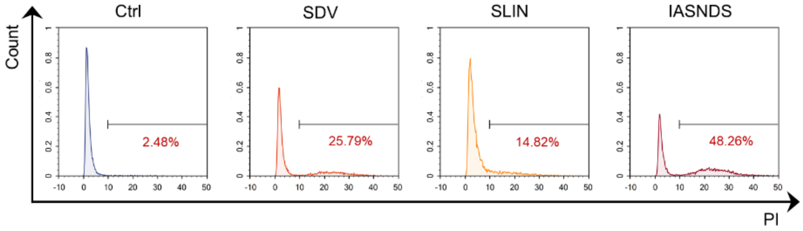

Supplementary Figure 11. Flow cytometry analysis of QL01#GBM cell staining under different treatment conditions (PI). (n = 6 independent experiments).

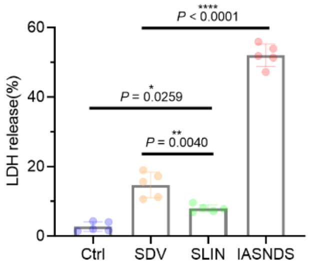

Supplementary Figure 12. LDH release analysis after 24 hours of PBS, SDV, SLIN, and IASNDS treatment in QL01#GBM cells. ($n = 5$ independent experiments). Data are presented as the mean ± S.D. Statistical comparisons were performed using one-way ANOVA with Tukey's post hoc test, with asterisks indicating significant differences (*$P < 0.05$, **$P < 0.01$, ***$P < 0.001$, ****$P < 0.0001$). (exact P value: $P = 9.89E\text{-}13$). Source data are provided as a Source Data file.

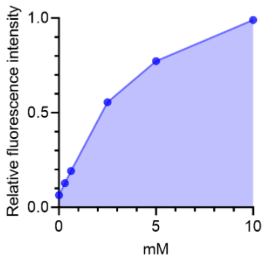

Supplementary Figure 13. Cumulative release of Cy5-modified CpG ODN at different ATP concentrations. ($n = 3$ independent experiments). Data are presented as the mean ± S.D. Source data are provided as a Source Data file.

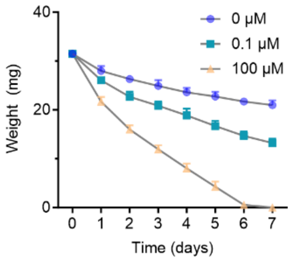

Supplementary Figure 14. Changes in gel weight after co-incubation of artificial cerebrospinal fluid with different concentrations of ATP and 30 uL of hydrogel for different periods of time. (n = 5 independent experiments). Source data are provided as a Source Data file.

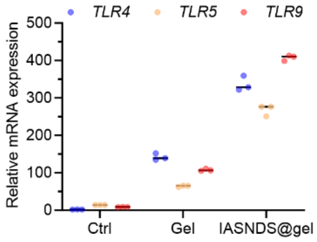

Supplementary Figure 15. The change in cellular expression of TLRs after 24 hours of coincubation of THP-1 macrophages with a hydrogel containing CpG ODN (Gel, IASNDS@gel). Data are presented as the mean ± S.D. (n = 3 independent experiments). Source data are provided as a Source Data file.

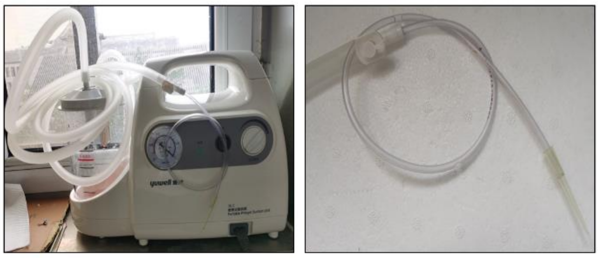

Supplementary Figure 16. Suction device for mouse GBM resection, simulating clinical surgical suctioning with specified parameters.

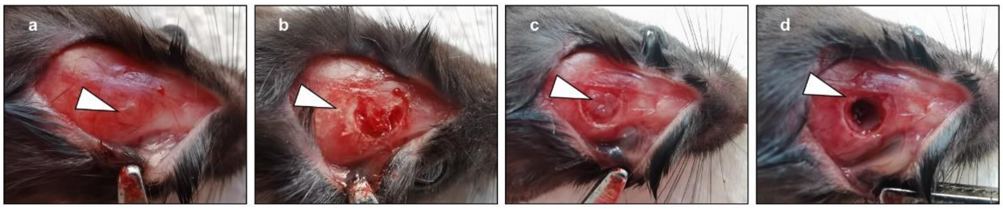

Supplementary Figure 17. Photographs illustrating the process of GBM resection with a combination of surgery and a suction device, white arrow shows tumor cavity.

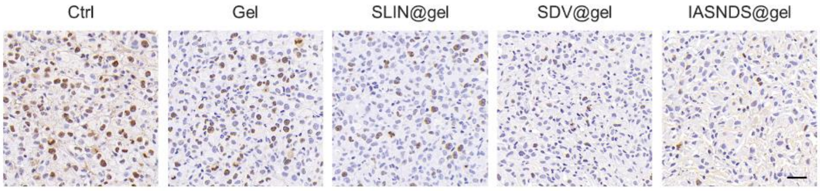

Supplementary Figure 18. Ki-67 immunohistochemical staining of tumor tissues in different groups. Representative images of the independent biological samples. (*n* = 3 independent biological samples). The scale bar is 10 μm.

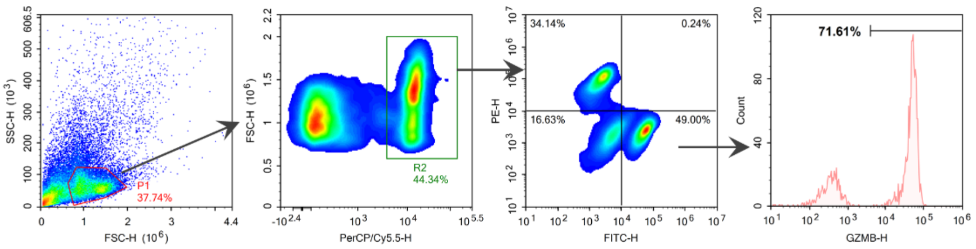

Supplementary Figure 19. Gating strategy for T-cell analysis. On day 20 post-treatment, groups of mice were humanely euthanized. Brain tissue samples were carefully collected and processed into cell suspensions using a glass grinder. Following the subsequent centrifugation and filtration steps, individual cells were quantified for flow cytometry analysis of T lymphocytes. A total of 1,000,000 cellular events was acquired for each sample during the analysis. Relevant cells were identified based on their locations within the FSC/SSC plot, which took into account their size. The total number of T cells was then determined by focusing on $CD3^+$ cell counts. Furthermore, within the population of $CD3^+$ T lymphocytes, a subset consisting of $CD4^+$ and $CD8^+$ T cells was carefully identified, as shown in Figure 7b. In the final step, a thorough analysis was conducted to identify granzyme B-positive T lymphocytes within the $CD8^+$ T lymphocytes, as shown in Figure 7e.

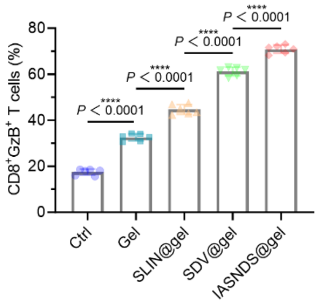

Supplementary Figure 20. Statistical analysis of $CD3^{+}CD8^{+}$Granzyme B^{+} T cells in GBM tissues under various treatment conditions. ($n = 6$ independent experiments). Data are presented as the mean ± S.D. Statistical comparisons were performed using one-way ANOVA with Tukey's post hoc test, with asterisks indicating significant differences. (exact P values: Ctrl vs. Gel $P = 6.32E-13$, Gel vs. SLIN@gel $P = 8.0463E-11$, SLIN@gel vs. SDV@gel $P = 1.14E-13$, SDV@gel vs. IASNDS@gel $P = 7.48308E-09$); ****$P < 0.0001$. Source data are provided as a Source Data file.

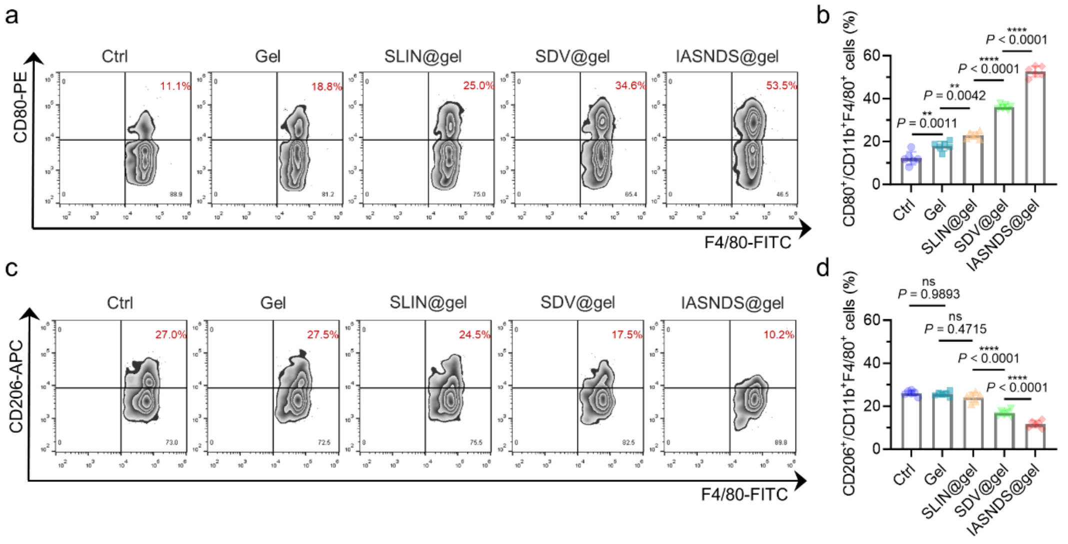

Supplementary Figure 21. Flow cytometric results (a) and analysis of statistical differences (b) in intra-tumoral M1 macrophage cells in mice after treatment of in situ hormonal mice with different drugs. (*n* = 6 independent experiments). Flow cytometric results (c) and analysis of statistical differences (d) in intra-tumoral M2 macrophage cells in mice after treatment of in situ hormonal mice with different drugs. (*n* = 6 independent experiments). Statistical comparisons were performed using one-way ANOVA with Tukey's post hoc test, with asterisks indicating significant differences (ns = no significance, **P* < 0.05, ***P* < 0.01, ****P* < 0.001, *****P* < 0.0001). (exact *P* values of b: SLIN@gel vs. SDV@gel *P* = 1.18872E-09, SDV@gel vs. IASNDS@gel *P* = 1.0138E-11; exact *P* values of d: SLIN@gel vs. SDV@gel *P* = 2.20199E-07, SDV@gel vs. IASNDS@gel *P* = 8.00699E-05). Source data are provided as a Source Data file.

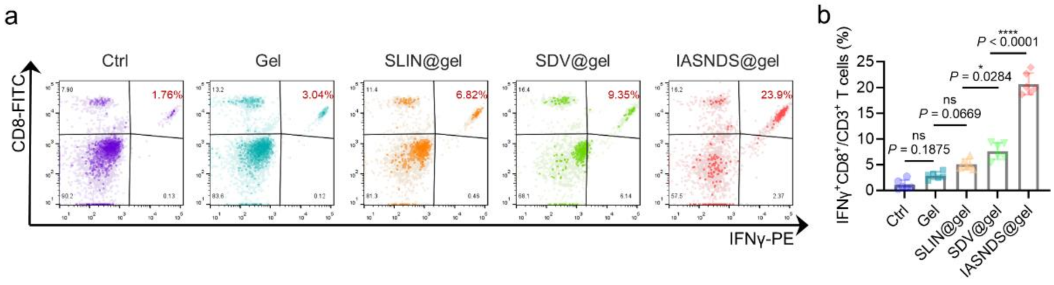

Supplementary Figure 22. (a) Flow cytometric analysis of $CD8^{+}IFN\gamma^{+}$ cells in different treatment groups of mice and analysis of statistical results (b). ($n = 6$ independent experiments). Statistical comparisons were performed using one-way ANOVA with Tukey's post hoc test, with asterisks indicating significant differences (ns = no significance, *$P < 0.05$, ****$P < 0.0001$). (exact P value: SDV@gel vs. IASNDS@gel $P =$ 8.7E-14). Source data are provided as a Source Data file.

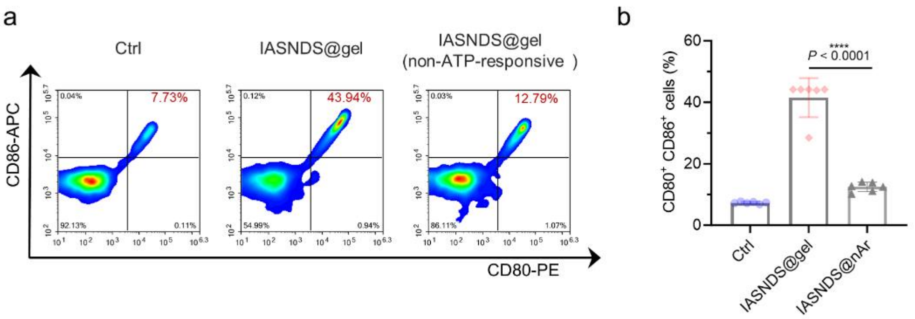

Supplementary Figure 23. (a) Flow cytometric analysis of APCs within ATP-responsive and non-responsive gels. (b) Shows statistical differences in $CD80^{+}CD86^{+}$ cells. ($n = 6$ independent experiments). (exact P value: IASNDS@gel vs. IASNDS@nAr $P = 3.20917E\text{-}09$); $^{****}P < 0.0001$. Source data are provided as a Source Data file.

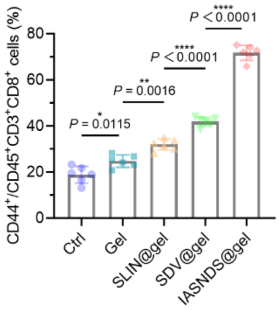

Supplementary Figure 24. Differences of immune memory cells in tumor tissues of mice in different treatment groups. ($n = 6$ independent experiments). Statistical comparisons were performed using one-way ANOVA with Tukey's post hoc test, with asterisks indicating significant differences (*$P < 0.05$, **$P < 0.01$, ****$P < 0.0001$). (exact P values: SLIN@gel vs. SDV@gel $P =$ 2.5305E-05, SDV@gel vs. IASNDS@gel $P =$ 3.6E-14). Source data are provided as a Source Data file.

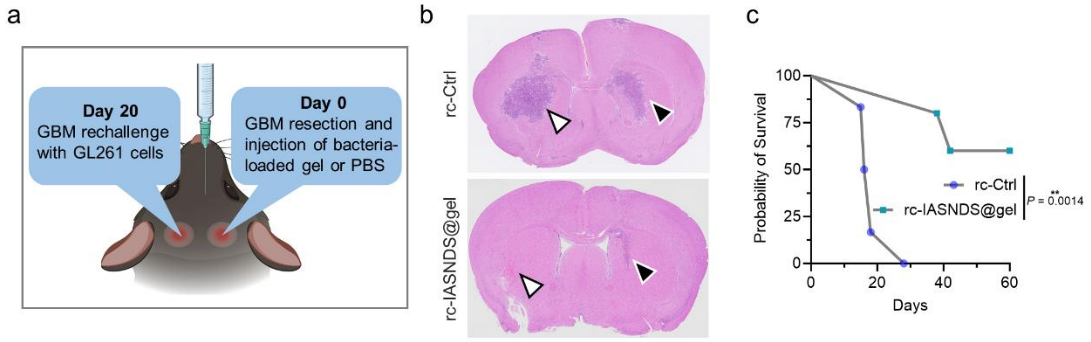

Supplementary Figure 25. Rechallenge the experiment. (a) Diagram of the experimental design model. (b) H&E staining of mouse brain tissue sections. ($n = 3$ independent biological samples). (c) Kaplan-Meier survival analysis of rechallenged mice. ($n = 6$ independent biological samples). Data analysis was performed employing the log-rank (Mantel–Cox) test. Source data are provided as a Source Data file.

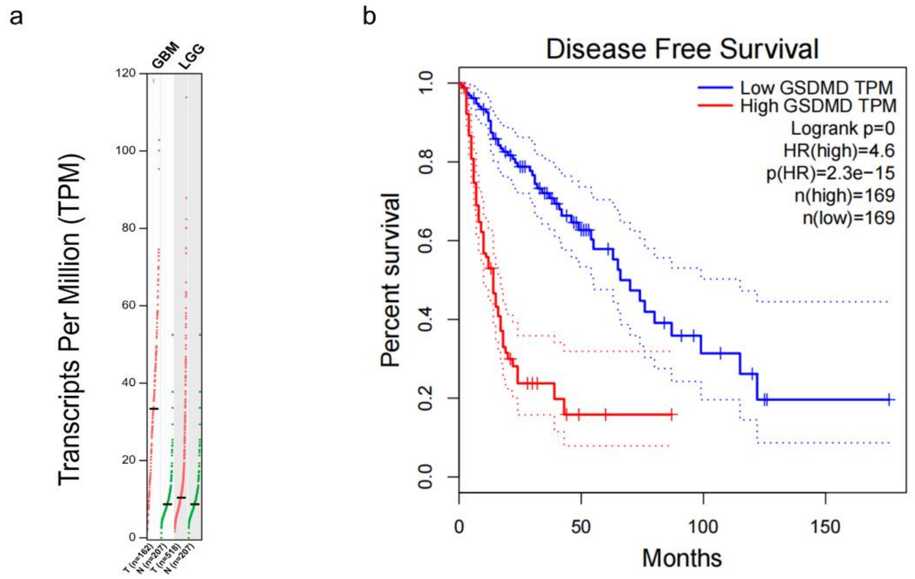

Supplementary Figure 26. (a) GEPIA database analysis of *GSDMD* mRNA differences between GBM and LGG. (b) Difference in survival time analyzed between patients with high and ground expression of *GSDMD*.

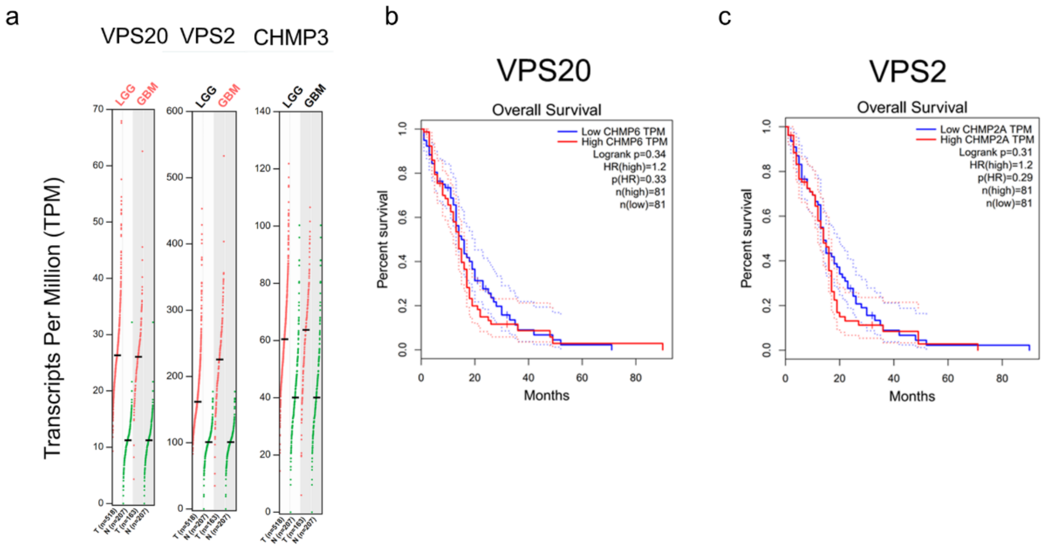

Supplementary Figure 27. (a) We analyzed the expression of ESCRT-III complex-related genes (*VPS20*, *VPS2*, *CHMP3*) in tumor samples from GBM and LGG patients. (b) Difference between *VPS20* expression and patient prognosis. (c) Difference between *VPS2* expression and patient prognosis.

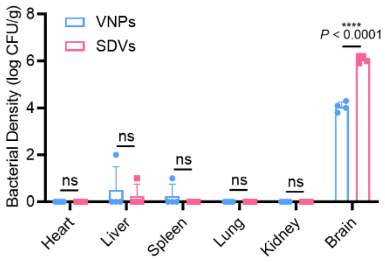

Supplementary Figure 28. Tail vein injection of VNP20009 and SDVs for 2 hours was followed by extraction and bacterial culture of mouse heart, liver, spleen, lung, kidney and brain tissue suspensions. ($n = 4$ independent experiments). The statistical comparisons were performed with two-tailed, unpaired Student's t tests. (exact P value: SDVs vs. VNPs $P =$ 4.09991E-06); ****$P < 0.0001$. Source data are provided as a Source Data file.

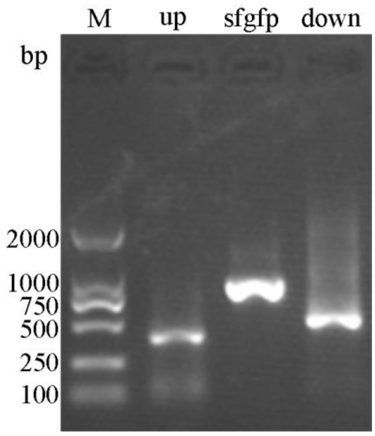

Supplementary Figure 29. Agarose gel electrophoresis assay of invA-up-sfgfp-down amplification. ($n = 3$ independent experiments). M refers to DL2000 DNA marker, up refers to the amplified upstream homology arm fragment, sfgfp is the amplified *sfgfp* fragment with promoter, and down refers to the amplified downstream homology arm fragment.

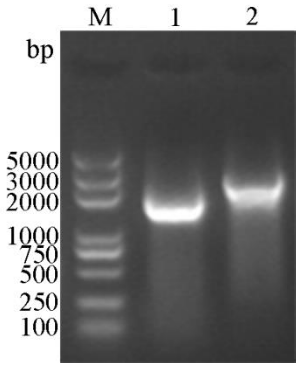

Supplementary Figure 30. Agarose gel electrophoresis assay of identification of wild bacteria and integrating bacteria. (*n* = 3 independent experiments). 1 refers to the amplification product of VNP20009 Δ*invA:sfgfp*. 2 refers to the amplification product of the original VNP20009, and the 1 product was sent for sequencing.

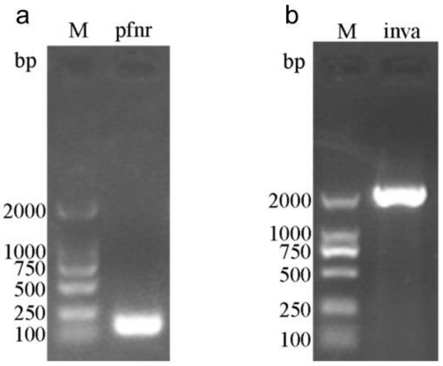

Supplementary Figure 31. Agarose gel electrophoresis detection of *FNR* promoter fragment (a) and *invA* gene amplification (b). ($n = 3$ independent experiments).

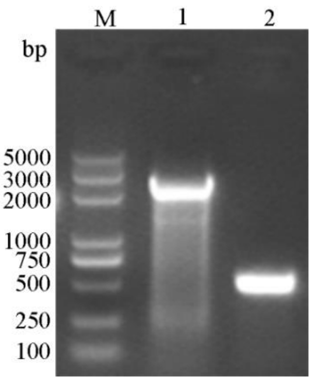

Supplementary Figure 32. Agarose gel electrophoresis assay of identification of FNR-invA integration. ($n = 3$ independent experiments). 1 refers to VNP20009 Δ*invA:sfgfp glms:FNR-invA* amplification product. 2 refers to VNP20009 original bacterial amplification product.

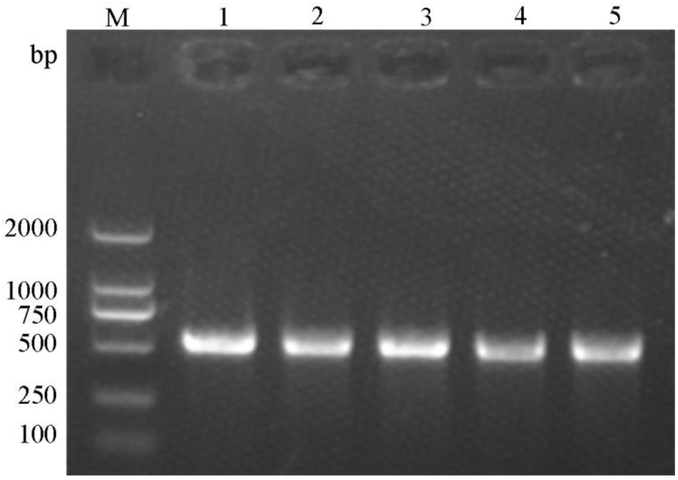

Supplementary Figure 33. PCR identification of colonies of VNP20009 Δ*invA:sfgfp glms:FNR-invA pBAD-LysE*. (*n* = 3 independent experiments). 1-5 were randomly picked clone numbers

Time (Day)	Ctrl (CFU/mL)		IASNDS@gel (CFU/mL)	
	Blood	CSF	Blood	CSF
1	0	0	0	1
3	0	0	0	0
5	0	0	0	0.5
7	0	0	0	0
9	0	0	0	0

Supplementary Table 1. Bacterial culture of cerebrospinal fluid and blood at different time points after local injection of IASNDS@gel in the operative cavity and counting of colonies ($n = 5$ independent experiments).

Supplementary Table 2.

ATP Aptamer-NH_2 (5'to 3')	NH_2-ACCTGGGGGAGTATTGCGGAGGAAGGT
ATP Aptamer-BHQ3	ACCTGGGGGAGTATTGCGGAGGAAGGT-BHQ3
CpG-cApt	TCGTCGTTTTCGGCGCGCGCCGACCTTCCTCCGCAA
CpG-cApt-Cy5	Cy5-TCGTCGTTTTCGGCGCGCGCCGACCTTCCTCCGCAA
CpG-cApt-Cy5.5	Cy5.5-ACCTTCCTCCGCAA

Supplementary Table 3.

invA-up-F	TTCAGCGATATCCAAATGTTGCA
invA-sfgfp-R	CCGATGATTAATTGTCAAGCGAATTACGAGCAGTAATGGT
invA-sfgfp-F	GATGAGCTCTACAAATAGATCCTGTTAATATTAAATTAAG
invA-down-R	GATATTGACGCGAATGACGCCAG
sfgfp-invA-F	ACTGCTCGTAATTCGCTTGACAATTAATCATCGGCTCGTA
sfgfp-invA-R	ATTTAATATTAACAGGATCTATTTGTAGAGCTCATCCATG
invA-19-F	TGTAAAACGACGGCCAGTTTCAGCGATATCCAAATGTTGCA
invA-19-R	CTATGACCATGATTACGCCGATATTGACGCGAATGACGCCAG
invA-ter-F	GCTCCACGAATATGCTCCACAAG
invA-ter-R	TAACAGTGCTCGTTTACGACCTG
glms-19-F	TGTAAAACGACGGCCAGTCGCGGAACAACTGGTCAGGGAAA
glms-19-R	CTATGACCATGATTACGCCAAGCATCACGCGCTATTCCTGG
glms-FNR-R	AAACTTTGCGGCGTTTTTGTACAAATGTAGTACCAGGCAG
invA-FNR-F	AAGGAGATATACATATGGTGCTGCTTTCTCTACTTAACAG
invA-his-R	GTGGTGGTGGTGGTGGTGTATTGTTTTTATAACATTCACT
glms down-invA-F	CACCACCACCACCACCACTAATTACTCTACGGTAACCGATTTC
glms-up-F	CGCGGAACAACTGGTCAGGGAAA
glms-down-R	TCTTCGCCGATCAGGATGCGGGT
pBAD-F	TAATCACGGCAGAAAAGTCCA
pBAD-R	AATCTTCTCTCATCCGCCAAA
