# Supplementary material for: Stimulation of tumoricidal immunity via bacteriotherapy inhibits glioblastoma relapse
Source: Nat Commun. 2024 May 18;15:4241. doi: 10.1038/s41467-024-48606-5 (PMC11102507; doi:10.1038/s41467-024-48606-5)
Supplement: Supplementary file 3 — Reporting Summary [file 41467_2024_48606_MOESM3_ESM.pdf]

nature portfolio

Corresponding author(s): Chen Chen, Xinyi Jiang, Shilei Ni

Last updated by author(s): Apr 28, 2024

Reporting Summary

Nature Portfolio wishes to improve the reproducibility of the work that we publish. This form provides structure for consistency and transparency in reporting. For further information on Nature Portfolio policies, see our Editorial Policies and the Editorial Policy Checklist.

Statistics

For all statistical analyses, confirm that the following items are present in the figure legend, table legend, main text, or Methods section.

n/a	Confirmed	
☐	☒	The exact sample size (*n*) for each experimental group/condition, given as a discrete number and unit of measurement
☐	☒	A statement on whether measurements were taken from distinct samples or whether the same sample was measured repeatedly
☐	☒	The statistical test(s) used AND whether they are one- or two-sided<br>*Only common tests should be described solely by name; describe more complex techniques in the Methods section.*
☐	☒	A description of all covariates tested
☐	☒	A description of any assumptions or corrections, such as tests of normality and adjustment for multiple comparisons
☐	☒	A full description of the statistical parameters including central tendency (e.g. means) or other basic estimates (e.g. regression coefficient) AND variation (e.g. standard deviation) or associated estimates of uncertainty (e.g. confidence intervals)
☐	☒	For null hypothesis testing, the test statistic (e.g. *F*, *t*, *r*) with confidence intervals, effect sizes, degrees of freedom and *P* value noted<br>*Give P values as exact values whenever suitable.*
☒	☐	For Bayesian analysis, information on the choice of priors and Markov chain Monte Carlo settings
☒	☐	For hierarchical and complex designs, identification of the appropriate level for tests and full reporting of outcomes
☒	☐	Estimates of effect sizes (e.g. Cohen's *d*, Pearson's *r*), indicating how they were calculated

Our web collection on statistics for biologists contains articles on many of the points above.

Software and code

Policy information about availability of computer code

Data collection	Applied Biosystems7900 Real-Time PCR system, BD FACSCelesta, NovoCyte Quanteon 4020, Beckman Coulter Moflo Astrios EQ, Carl Zeiss LSM780, PerkinElmer IVIS Spectrum, Olympus, VERSUS120, ChemDraw Ultra 7.0, Mercury Plus 400 or 500 spectrometer, Thermo Finnigan LCQ DECA XP spectrometer, Varian Cary 300.
Data analysis	Statistical analysis was performed on Graphpad Prism 10.1.2, flow cytometry data were analyzed on NovoExpress (1.6.1) software, confocol images were analyzed by ImageJ software (1.52v) .

For manuscripts utilizing custom algorithms or software that are central to the research but not yet described in published literature, software must be made available to editors and reviewers. We strongly encourage code deposition in a community repository (e.g. GitHub). See the Nature Portfolio guidelines for submitting code & software for further information.

Data

Policy information about availability of data

All manuscripts must include a data availability statement. This statement should provide the following information, where applicable:

- Accession codes, unique identifiers, or web links for publicly available datasets
- A description of any restrictions on data availability
- For clinical datasets or third party data, please ensure that the statement adheres to our policy

Source data are provided with this paper. The remaining data are available within the Article, Supplementary Information or Source Data file.

Research involving human participants, their data, or biological material

Policy information about studies with human participants or human data. See also policy information about sex, gender (identity/presentation), and sexual orientation and race, ethnicity and racism.

Reporting on sex and gender	N/A
Reporting on race, ethnicity, or other socially relevant groupings	N/A
Population characteristics	N/A
Recruitment	N/A
Ethics oversight	N/A

Note that full information on the approval of the study protocol must also be provided in the manuscript.

Field-specific reporting

Please select the one below that is the best fit for your research. If you are not sure, read the appropriate sections before making your selection.

- [x] Life sciences
- [ ] Behavioural & social sciences
- [ ] Ecological, evolutionary & environmental sciences

For a reference copy of the document with all sections, see nature.com/documents/nr-reporting-summary-flat.pdf

Life sciences study design

All studies must disclose on these points even when the disclosure is negative.

Sample size	Sample size are provided in the figure legends for each experiment and reasonable sample sizes were chosen to ensure they are sufficient for statistical comparison between different groups. For in vitro experiments and analysis, a sample size of 3 was used to detect a significant difference between different groups. For analysing the anti-GBM effects in vivo, 10 mice from each group were housed for bioluminescence imaging, 3 mice from each group were euthanized for histological staining. 10 mice from each group were housed for Kaplan–Meier survival curves. The in vivo mechanisms of anti-GBM studies were performed with 6 mice per group. Details regarding sample size of all experiments are provided in the Methods section and figure legends. Samples sizes for in vivo experiments were chosen empirically based upon preliminary therapy experiments. Input and approval from the Research Ethics Committee of Shandong University and the Ethics Committee of Qilu Hospital (Shandong, China) were also considered. Sample sizes for in vitro experiments were also chosen empirically based upon preliminary experiments to achieve statistical significance.
Data exclusions	No animals and/or data were excluded.
Replication	Experiments were replicated independently for at least 3 times. The number of replicates is detailed in the caption of each figures in the main manuscript and supplementary information files.
Randomization	The dosing groups were filled by random selection from the same pool of animals for in vivo experiments. Groups in all the in vitro and in vivo experiments were selected randomly. Organisms were cultured and maintained in the same environment and randomly allocated to each group.
Blinding	All the investigators were blinded to group allocation during data collection and analysis.

Reporting for specific materials, systems and methods

We require information from authors about some types of materials, experimental systems and methods used in many studies. Here, indicate whether each material, system or method listed is relevant to your study. If you are not sure if a list item applies to your research, read the appropriate section before selecting a response.

Materials & experimental systems

n/a	Involved in the study
☐	☒ Antibodies
☐	☒ Eukaryotic cell lines
☒	☐ Palaeontology and archaeology
☐	☒ Animals and other organisms
☒	☐ Clinical data
☒	☐ Dual use research of concern
☒	☐ Plants

Methods

n/a	Involved in the study
☒	☐ ChIP-seq
☐	☒ Flow cytometry
☒	☐ MRI-based neuroimaging

Antibodies

Antibodies used

CD3-PerCP-Cy5.5 (BioLegend, Catalog number: 100218; Clone name: 17A2; 1:20 dilution);
CD4-PE (BioLegend, Catalog number: 100408; Clone name: GK1.5; 1:100 dilution);
CD8-FITC (BioLegend, Catalog number: 100706; Clone name: 53-6.7; 1:50 dilution);
Granzyme B-Alexa Fluor 647 (BioLegend, Catalog number: 372220; Clone name: QA16A02; 1:20 dilution);
IFNγ-PE (Invitrogen, Catalog number: 12-7319-41; Clone name: 4S.B3; 1:20 dilution);
CD80-PE (BioLegend, Catalog number: 104708; Clone name: 16-10A1; 1:40 dilution);
CD86-APC (BioLegend, Catalog number: 105012; Clone name: GL-1; 1:80 dilution);
CD206-APC (BioLegend, Catalog number:141708; Clone name: C068C2; 1:40 dilution);
F4/80-FITC (BioLegend, Catalog number:123108; Clone name: BM8; 1:200 dilution);
β-Actin (Cell Signaling Technology, Catalog number: 4970S; Clone name: 13E5; 1:1000 dilution);
Calnexin (Cell Signaling Technology, Catalog number: 2679S; Clone name: C5C9; 1:1000 dilution);
Gasdermin D (Cell Signaling Technology, Catalog number: 39754S; Clone name: E9S1X; 1:1000 dilution);
GSDMD-N (Cell Signaling Technology, Catalog number: 36425S; Clone name: Asp275; 1:1000 dilution);
Cleaved Caspase-1 (Cell Signaling Technology, Catalog number: 4199T; Clone name: Asp297; 1:1000 dilution);
TSG101 (Abcam, Catalog number: ab125011; Clone name: EPR7130; 1:1000 dilution).

Validation

All antibodies were verified by the supplier has been quality tested. All the antibodies used are from commercial sources and have been validated by the vendors. Validation data are available on the manufacturer's website.
1. CD3-PerCP-Cy5.5 has been validated to be used for flow cytometric analysis and mentioned species reactivity with mouse. (https://www.biolegend.com/en-gb/products/percp-cyanine5-5-anti-mouse-cd3-antibody-5596)
2. CD4-PE has been validated to be used for flow cytometric analysis and mentioned species reactivity with mouse. (https://www.biolegend.com/en-gb/products/pe-anti-mouse-cd4-antibody-250)
3. CD8-FITC has been validated to be used for flow cytometric analysis and mentioned species reactivity with mouse. (https://www.biolegend.com/en-gb/products/fitc-anti-mouse-cd8a-antibody-153)
4. Granzyme B-Alexa Fluor 647 has been validated to be used for flow cytometric analysis and mentioned species reactivity with mouse. (https://www.biolegend.com/en-gb/products/alexa-fluor-647-anti-humanmouse-granzyme-b-recombinant-antibody-15616)
5. IFNγ-PE has been validated to be used for flow cytometric analysis and mentioned species reactivity with mouse. (https://www.thermofisher.cn/cn/zh/antibody/product/IFN-gamma-Antibody-clone-4S-B3-Monoclonal/12-7319-41)
6. CD80-PE has been validated to be used for flow cytometric analysis and mentioned species reactivity with mouse. (https://www.biolegend.com/en-gb/products/pe-anti-mouse-cd80-antibody-43)
7. CD86-APC has been validated to be used for flow cytometric analysis and mentioned species reactivity with mouse. (https://www.biolegend.com/en-gb/products/apc-anti-mouse-cd86-antibody-2896)
8. CD206-APC has been validated to be used for flow cytometric analysis and mentioned species reactivity with mouse. (https://www.biolegend.com/en-gb/products/apc-anti-mouse-cd206-mmr-antibody-7425)
9. F4/80-FITC has been validated to be used for flow cytometric analysis and mentioned species reactivity with mouse. (https://www.biolegend.com/en-gb/products/fitc-anti-mouse-f4-80-antibody-4067)
10. β-Actin has been validated to be used for western blotting and mentioned species reactivity with human/mouse. (https://www.cellsignal.cn/products/primary-antibodies/b-actin-13e5-rabbit-mab/4970)
11. Calnexin has been validated to be used for western blotting and mentioned species reactivity with human. (https://www.cellsignal.cn/products/primary-antibodies/calnexin-c5c9-rabbit-mab/2679)
12. Gasdermin D has been validated to be used for western blotting and mentioned species reactivity with human/mouse. (https://www.cellsignal.cn/products/primary-antibodies/gasdermin-d-e9s1x-rabbit-mab/39754)
13. GSDMD-N has been validated to be used for western blotting and mentioned species reactivity with human. (https://www.cellsignal.cn/products/primary-antibodies/cleaved-gasdermin-d-asp275-e7h9g-rabbit-mab/36425)
14. Cleaved Caspase-1 has been validated to be used for western blotting and mentioned species reactivity with human. (https://www.cellsignal.cn/products/primary-antibodies/cleaved-caspase-1-asp297-d57a2-rabbit-mab/4199)
15. TSG101 has been validated to be used for western blotting and mentioned species reactivity with human/mouse. (https://www.abcam.cn/products/primary-antibodies/tsg101-antibody-epr7130b-ab125011.html)

Eukaryotic cell lines

Policy information about cell lines and Sex and Gender in Research

Cell line source(s)

The mouse GBM cell line GL261 was acquired from the Cell Bank of the Chinese Academy of Sciences (Shanghai, China), while

	megakaryocytes (MEG01) were obtained from the American Type Culture Collection. The QL01#GBM cells that were surgically propagated from primary human glioblastoma were derived from a 56-year-old female patient, the postoperative pathological findings confirmed glioblastoma, the patient was underwent surgical resection at the Department of Neurosurgery, Qilu Hospital, Shandong University.
Authentication	A short tandem repeat DNA profiling method was used to authenticate the cell lines and the results were compared with reference database. Moreover, stable luciferase expression GL261 cell line was authenticated by in vivo imaging system to evaluate the luminescence intensity.
Mycoplasma contamination	All cell lines were tested for mycoplasma contamination and no mycoplasma contamination was found.
Commonly misidentified lines (See ICLAC register)	No commonly misidentified cell lines were used.

Animals and other research organisms

Policy information about studies involving animals; ARRIVE guidelines recommended for reporting animal research, and Sex and Gender in Research

Laboratory animals	C57BL/6 mice (male, 6 weeks) were ordered from GemPharmatech Co., Ltd. and housed in a specific-pathogen-free animal facility at ambient temperature (22 ± 2 °C), air humidity 40%–70% and 12-h dark/12-h light cycle.
Wild animals	No wild animal was used in this study.
Reporting on sex	This study was not a sex-based study, sex was not a variable in this study where all mice were males.
Field-collected samples	The study did not involve samples collected from field.
Ethics oversight	All procedures were approved by the Research Ethics Committee of Shandong University and the Ethics Committee of Qilu Hospital (Shandong, China), in compliance with all relevant ethical regulations. According to the ethics committee, the size of the tumor must not exceed 10% of the animal's body weight, while the animal must not lose more than 20% of its body weight during the research. The maximal tumor burden was not exceeded in this study.

Note that full information on the approval of the study protocol must also be provided in the manuscript.

Plants

Seed stocks	*Report on the source of all seed stocks or other plant material used. If applicable, state the seed stock centre and catalogue number. If plant specimens were collected from the field, describe the collection location, date and sampling procedures.*
Novel plant genotypes	*Describe the methods by which all novel plant genotypes were produced. This includes those generated by transgenic approaches, gene editing, chemical/radiation-based mutagenesis and hybridization. For transgenic lines, describe the transformation method, the number of independent lines analyzed and the generation upon which experiments were performed. For gene-edited lines, describe the editor used, the endogenous sequence targeted for editing, the targeting guide RNA sequence (if applicable) and how the editor was applied.*
Authentication	*Describe any authentication procedures for each seed stock used or novel genotype generated. Describe any experiments used to assess the effect of a mutation and, where applicable, how potential secondary effects (e.g. second site T-DNA insertions, mosiacism, off-target gene editing) were examined.*

Flow Cytometry

Plots

Confirm that:

- [x] The axis labels state the marker and fluorochrome used (e.g. CD4-FITC).
- [x] The axis scales are clearly visible. Include numbers along axes only for bottom left plot of group (a 'group' is an analysis of identical markers).
- [x] All plots are contour plots with outliers or pseudocolor plots.
- [x] A numerical value for number of cells or percentage (with statistics) is provided.

Methodology

Sample preparation	Each group of mice was euthanized on day 20 post-treatment. Tumor tissue was collected, processed, filtered in complete RPMI-1640 using a 70 μm cell filter, centrifuged, and lysed in ACK lysis buffer for red blood cells. Cells were lysed in ACK lysis buffer. Single cell suspensions were obtained and stained with antibodies according to the manufacturer's protocol , and then analyzed by flow cytometry.
Instrument	NovoCyte Quanteon 4020

Software	NovoExpress 1.6.1
Cell population abundance	The cell abundance was shown in the results.
Gating strategy	In general, cells were first gated on FSC/SSC. Singlet cells were gated using FSC-H and FSC-A. For CD3+CD4+ and CD3+CD8+ T cells analysis, flow cytometry measurement was first gated with CD3+, then CD4+ and CD8+ T cells were gated with CD4 and CD8. For CD3+CD8+granzyme B+ T cells analysis, flow cytometry measurement was first gated with CD3, then gated with CD8 + and granzyme B. For the analysis of immune memory cells, flow cytometry measurement was first gated with CD45+, then gated with CD3+ and CD8+, and finally the number of CD44+ cells was analyzed. For CD3+CD8+IFNγ+ T cells analysis, flow cytometry measurement was first gated with CD3, then gated with CD8+ and IFNγ+. For the analysis of M1 macrophages and M2 macrophages, flow cytometry measurement was first gated with CD11b+ and F4/80+, then gated with CD80+ or CD206+.

☒ Tick this box to confirm that a figure exemplifying the gating strategy is provided in the Supplementary Information.
